# VTET: a variable threshold exact test for identifying disease-associated copy number variations enriched in short genomic regions

**DOI:** 10.3389/fgene.2014.00053

**Published:** 2014-03-18

**Authors:** Jianxin Shi, Xiaohong R. Yang, Neil E. Caporaso, Maria T. Landi, Peng Li

**Affiliations:** Division of Cancer Epidemiology and Genetics, National Cancer Institute, National Institutes of HealthBethesda, MD, USA

**Keywords:** copy number varination, variable threshold exact test, genome-wide association study, interval-based association test, lung cancer CNV analysis

## Abstract

Copy number variations (CNVs) constitute a major source of genetic variations in human populations and have been reported to be associated with complex diseases. Methods have been developed for detecting CNVs and testing CNV associations in genome-wide association studies (GWAS) based on SNP arrays. Commonly used two-step testing procedures work well only for long CNVs while direct CNV association testing methods work only for recurrent CNVs. Assuming that short CNVs disrupting any part of a given genomic region increase disease risk, we developed a variable threshold exact test (VTET) for testing disease associations of CNVs randomly distributed in the genome using intensity data from SNP arrays. By extensive simulations, we found that VTET outperformed two-step testing procedures based on existing CNV calling algorithms for short CNVs and that the performance of VTET was robust to the length of the genomic region. In addition, VTET had a comparable performance with CNVtools for testing the association of recurrent CNVs. Thus, we expect VTET to be useful for testing disease associations of both recurrent and randomly distributed CNVs using existing GWAS data. We applied VTET to a lung cancer GWAS and identified a genome-wide significant region on chromosome 18q22.3 for lung squamous cell carcinoma.

## Introduction

Copy number variations (CNVs) are one of the major sources of genetic variations in the human genome (Redon et al., 2006) and have been reported to be associated with a variety of complex diseases (Sebat et al., 2007; Consortium, 2008; Stefansson et al., 2008; Bucan et al., 2009; Diskin et al., 2009; Glessner et al., 2009; McCarthy et al., 2009; Levinson et al., 2011). In genome-wide association studies (GWAS) based on SNP arrays, CNVs are inferred based on two measurements at each probe in the SNP array: the Log R Ratio (LRR) and the B Allele Frequency (BAF). Identifying disease-causing rare CNVs helps to elucidate the etiology of complex diseases, improve risk prediction models and may contribute to personalized treatment in the future. However, detecting CNV associations from GWAS SNP arrays is computationally intensive and statistically challenging, particularly for short CNVs.

There are currently two strategies for testing CNV associations. As the standard approach, CNVs are called for each subject using CNV detection algorithms (Olshen et al., 2004; Colella et al., 2007; Wang et al., 2007; Korn et al., 2008; Coin et al., 2010) followed by the association analysis comparing each probe or genomic region against the disease phenotype of interest. This standard two-step strategy is most useful for detecting associations of long CNVs with excellent calling accuracy. In fact, the majority of the reported associations are based on long CNVs covering over 10 probes. However, a large proportion of germline CNVs are short and cover only a few probes in genotyping or array CGH platforms (Redon et al., 2006). The sensitivity of detecting short CNVs using these algorithms is typically low. Consequently, testing associations of short CNVs covering less than 10 probes is expected to have a low statistical power based on the standard two-step methods using these widely-used software packages. More algorithms have been recently developed with better sensitivity for detecting shorter CNVs (Pique-Regi et al., 2008; Wang et al., 2009; Jeng et al., 2010; Jang et al., 2013); however their performances for large-scale GWAS data remain to be systematically evaluated.

The second strategy is to directly test the CNV associations from the intensity data without making CNV calls (Barnes et al., 2008; Ionita-Laza et al., 2008; Eleftherohorinou et al., 2011; Shi and Li, 2013). The simplest method is to directly test the association for each probe using LRR as a surrogate (Ionita-Laza et al., 2008). This method does not use spatial information of CNVs or the distribution of the intensity data and thus is not expected to be efficient. CNVtools (Barnes et al., 2008) tests associations in known CNV regions based on a Gaussian mixture model. We have recently developed a method based on a hidden Markov model (Shi and Li, 2013) for both documented and undocumented CNVs in GWAS. These methods are fully efficient when CNVs are largely overlapped or recurrent with the same boundaries.

**Figure 1:**
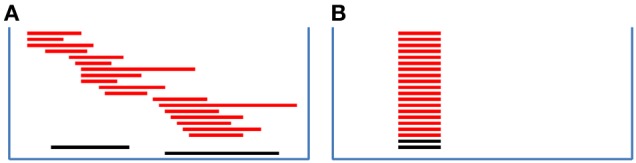
**Recurrent CNVs and randomly distributed CNVs in a given genomic region**. Each red (black) bar represents a CNV in a case (control). **(A)** shows CNVs with locations randomly distributed in the region and **(B)** shows recurrent CNVs with identical boundaries. In both figures, there are 18 cases carrying CNVs while two controls carrying CNVs. Assuming equal number of cases and controls, CNVs more frequently disrupt the genomic region in cases than in controls. When CNV status is known, the association can be tested using the Fisher's exact test.

In this manuscript, we consider the scenario that cases are more frequently disrupted by CNVs than controls in a given genomic region while CNVs are randomly distributed in the region with various boundaries (Figure 1), as shown as an example in a GWAS of autism (Glessner et al., 2009). The existing methods designed for testing the associations of overlapping CNVs (Barnes et al., 2008; Ionita-Laza et al., 2008; Eleftherohorinou et al., 2011; Shi and Li, 2013) are expected to perform poorly in this scenario. We developed a statistical framework, the variable threshold exact test (VTET), for testing CNV associations efficiently for this scenario. Briefly, VTET first evaluates the statistical evidence of carrying a CNV anywhere in the selected target region and then performs exact tests to evaluate the degree of genetic association using different thresholds to define tentative CNV carriers. The significance can be efficiently evaluated by permuting case-control labels. We show through extensive simulations with realistic settings that VTET performs very well even for short CNVs covered by as few as three probes and is much more powerful than the standard two-step testing procedures using widely-used CNV calling software packages, e.g., PennCNV (Wang et al., 2007) and circular binary segmentation (CBS) (Olshen et al., 2004). In addition, VTET performs comparably with CNVtools for recurrent CNVs. Thus, VTET can be used to detect associations of both overlapping and non-overlapping CNVs. We illustrate the application of VTET using a published lung cancer GWAS.

## Materials and methods

### Quantify the evidence of a CNV in a genomic region

Consider a case-control study with *m* cases and *n* controls. Each subject is genotyped at *T* probes in a given genomic region. We use *i* = 1, …,*m* to index cases and *i* = 1 + *m*, …,*m* + *n* to index controls. For subject *i*, let *X*_*it*_ be the LRR and *B*_*it*_ be the BAF for probe *t*. Here, the LRR measures the total intensity of the fluorescence used to label the probe in the assay and is an approximation of the total amount of DNA. LRR is expected to be zero when there is no copy number change. A large value of LRR indicates a duplication whereas a small value of LRR indicates a deletion. For each probe, we denote the two alleles as A and B. The BAF measures the proportion of the DNA attributable to the B allele. The distribution of BAFs is shown in Table 1. BAFs close to 1/3 and 2/3 are indicative of duplications. The unknown copy number status is denoted as *c*_*it*_ ∈ {0,1,2,3}. Here, we do not consider CNVs with more than 3 copies because they are rare in the population. LRRs are independent across probes given the copy number status. Each *X*_*it*_ is normalized to follow *N*(0,1) when *c*_*it*_ = 2. We are interested in testing whether cases are more likely to carry a CNV, a deletion or duplication or either type of CNVs, in a given short genomic region (Figure 1).

**Table 1:** **Distribution of the B Allele Frequencies (BAF) given the genotype and the copy number**.

**Genotype**	**Distribution of BAF**
Copy number = 0	*U*[0,1]
A, AA, AAA	0.5*I*_*b* = 0_ + *I*_*b* > 0_ϕ(*b*/η _1_)
B, BB, BBB	0.5*I*_*b* = 1_ + *I*_*b* < 0_ϕ((*b* – 1)/η _1_)
AB	ϕ((*b*-0.5)/η _2_)
AAB	ϕ((*b*-1/3)/η _2_)
ABB	ϕ((*b*-2/3)/η _2_)

ϕ is the density function of N(0,1).

For convenience, we illustrate our algorithm for detecting CNV associations without considering the BAF information. We will then extend the algorithm to incorporate the BAF information to improve the power, particularly for duplications. We only consider hemizygous deletions (denoted as CN1) and duplications with three copies (denoted as CN3), given that the sensitivity for detecting homozygous deletions (CN0) is almost one and germline duplications with copy number >3 are very rare. Briefly, our method consists of two steps. In the first step, we quantify the evidence that subject *i* carries a CNV anywhere in the region. In the second step, we test whether cases are more likely to carry CNVs based on a VTET.

We define a binary variable *E*_*i*_ = 1 if subject *i* carries a CNV anywhere in the interval and *E*_*i*_ = 0 otherwise. We are interested in CNVs covering at least *L*(≥ 3) probes. The log likelihood ratio statistic (Olshen et al., 2004) based only on LRRs for detecting a CNV in [a,b] is

(1) $$z_{ab}^{i}=\sum_{t\;=\;a}^{b}X_{it}/{\left[b-a+1\right]}^{1/2}$$

where *z*^*i*^_*ab*_ ~ *N*(0,1) if *c*_*ia*_ = … = *c*_*ib*_ = 2. To search for CNVs covering at least *L* probes in the region, we calculate

(2) $$U_{i}=\underset{L\leq b-a+1,a<b}{\max}|z_{ab}^{i}|.$$

Let *U*^0^_*i*_ be the observed statistic value. The evidence that the given region carries a CNV is quantified as a *p*-value

(3) $$p_{i}=P\left(U_{i}=\underset{L\leq b-a+1,a<b}{\max}|z_{ab}^{i}|>U_{i}^{0}|E_{i}=0\right).$$

A small value of *p*_*i*_ supports the existence of a CNV in the region.

When *T* is sufficiently large, we can use Siegmund's method based on the random walk theory (Siegmund, 1992) to derive a very accurate asymptotic approximation *p*_*i*_ ≈ 2*TU*^0^_*i*_ λ^−2^ϕ(*U*^0^_*i*_)[(*s*_1_ −1)*e*^*s*_1_^ − (*s*_2_ −1)*e*^*s*_2_^]/8 with λ = −0.583, *s*_1_ = 2λ*U*^0^_*i*_/$\sqrt{L-1}$, *s*_2_ = 2λ*U*^0^_*i*_/$\sqrt{T+1}$ and ϕ(·) as the density function for *N*(0,1). However, the approximation performs poorly when *T* ≤ 50. Thus, we have performed 10^6^ Monte Carlo simulations to approximate *p*-values as small as 10^−5^, which is sufficiently accurate in our procedure for testing CNV associations.

Similarly, we can quantify the evidence for carrying a CN1 deletion or CN3 duplication. We define

(4) $$U_{i-}=\underset{L\leq b-a+1,a<b}{\max}-z_{ab}^{i}$$

for detecting CN1 deletions and

(5) $$U_{i+}=\underset{L\leq b-a+1,a<b}{\max}z_{ab}^{i}$$

for detecting CN3 duplications. Let *U*^0^_*i*−_ and *U*^0^_*i*+_ be the observed test values for subject *i*. The *p*-values are then defined as

(6) $$p_{i-}=P\left(U_{i-}=\underset{L\leq b-a+1,a<b}{\max}-z_{ab}^{i}>U_{i-}^{0}|E_{i}=0\right)$$

for detecting CN1 deletions and

(7) $$p_{i+}=P\left(U_{i+}=\underset{L\leq b-a+1,a<b}{\max}z_{ab}^{i}>U_{i+}^{0}|E_{i}=0\right)$$

for detecting CN3 duplications. Again, *p*-values are approximated by Monte Carlo simulations.

### A variable threshold exact test

Given a set of *p*-values {*p*_1_, …, *p_*m*_*, *p*_*m* + 1_, …, *p*_*m* + *n*_} for *m* cases and *n* controls, we test whether cases are more likely to carry CNVs in the region. We need to determine which subjects carry CNV based on the *p*-values. For a given threshold *q*, subjects with *p*_*i*_ ≤ *q* are considered as tentative CNV carriers. We define *a*(*q*) and *b*(*q*) to be the numbers of tentative CNV carriers in cases and controls, respectively. The genetic association is tested using the Fisher's exact test with *p*-value denoted as *P*(*q*). Here, the *p*-value *P*(*q*) depends on the threshold *q*.

An inappropriate choice of *q* may lead to a loss of statistical power. Choosing a liberal threshold *q* results in many false CNV carriers while choosing a rigorous *q* misses many true CNV carriers. One reasonable choice is *q* = 2 / (*m* + *n*), under which we expect two false positive CNV carriers out of the *m* + *n* subjects. If *m* ≈ *n*, we would expect roughly one false positive CNV carriers in cases and controls respectively. To make statistical power more robust, we choose a series of thresholds (*q*_1_, …, *q*_*K*_) to derive the association *p*-values (*P*(*q*_1_), …,*P*(*q*_*K*_)) based on the Fisher's exact test. The overall statistic is then defined as

(8) $$Q=\underset{1\leq k\leq K}{\min}P(q_{k})$$

**Figure 2:**
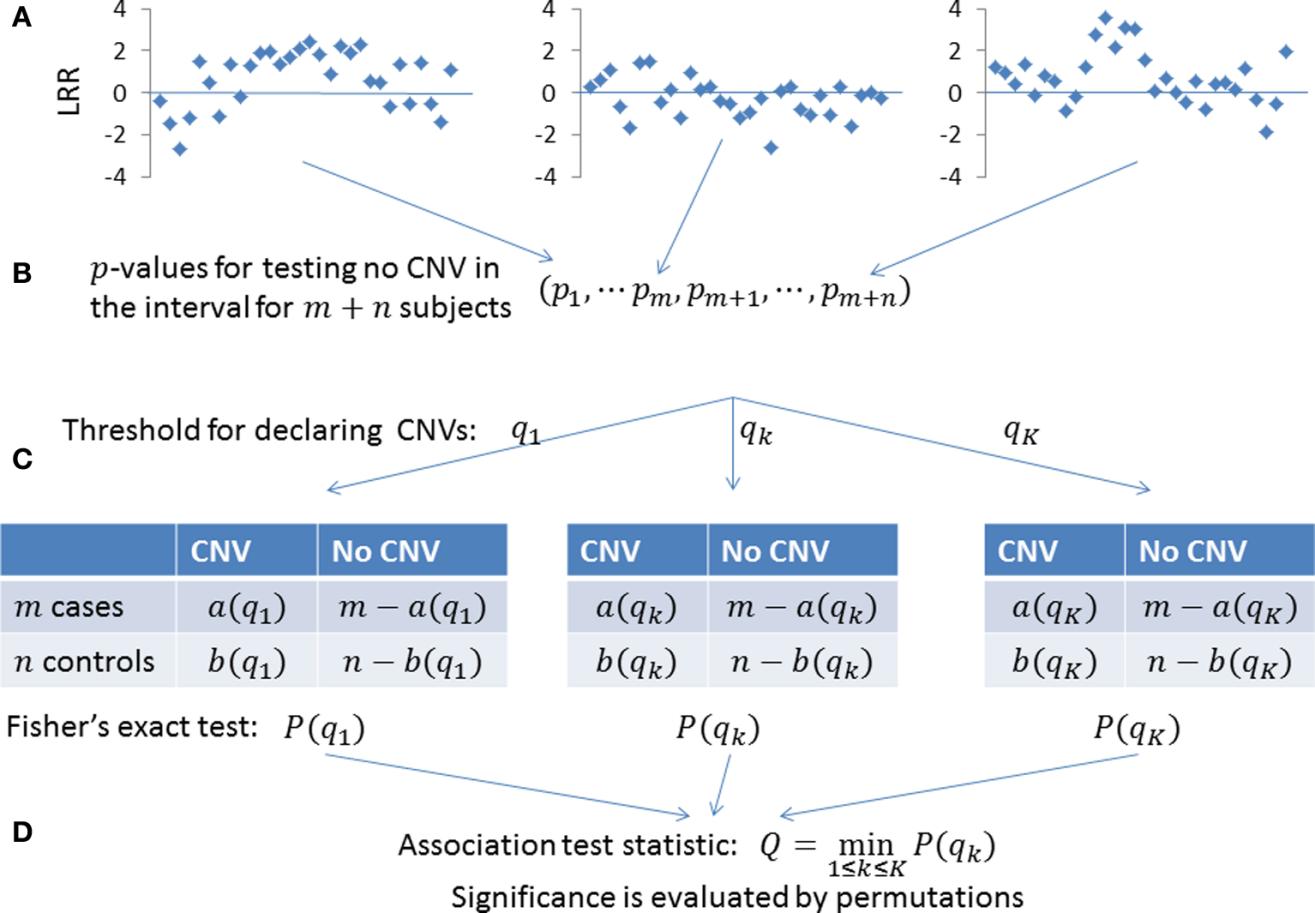
**Testing the association of CNVs in a given genomic region. (A)** LRRs in the given genomic region for *m* + *n* subjects. **(B)** The existence of a CNV anywhere in the region is quantified as a *p*-value. A small *p*-value indicates the presence of a CNV. **(C)** For a given threshold *q*_1_, subjects with *p*-value < *q*_1_ are determined as tentative CNV carriers. We have *a*(*q*_1_) CNV carriers in *m* cases and *b*(*q*_1_) CNV carriers in *n* controls, with the association tested by the Fisher's exact test. To make the power robust, we use multiple thresholds (*q*_1_, …, *q*_*K*_) to derive association *p*-values (*P*(*q*_1_), …,*P*(*q*_*K*_)). **(D)** The overall statistics is defined as the minimum of (*P*(*q*_1_), …,*P*(*q*_*K*_)) and its significance is determined by permuting the case-control status.

The significance is evaluated by permuting case-control status. In our implementation, we use *K* = 5 and choose (*q*_1_, …, *q*_5_) to expect (5, 2, 1, 0.5, 0.1) false CNV carriers, respectively. The procedure is summarized in Figure 2. We call the method as a VTET.

### Incorporating the baf information

Incorporating BAFs can substantially improve the sensitivity of detecting CN3 duplications (Shi and Li, 2012). Briefly, BAFs close to 1/3 or 2/3 support CN3 duplications while BAF close to 0 or 1 are not informative for the inference of CN3 duplications. For an informative BAF *b*_*it*_ ∈ [0.2,0.8], we convert *b*_*it*_ into a normal quantile *Y*_*it*_. Here, *Y*_*it*_ ~ *N*(0,1) if *c*_*it*_ = 2 and *Y*_*it*_ is large when *c*_*it*_ = 3. In addition, (*Y*_*i*1_, …, *Y*_*iT*_) are mutually independent for subject *i*. Details can be found in (Shi and Li, 2012). Based on simulations, we found that *cor*(*X*_*it*_, *Y*_*it*_) = −0.05 if *c*_*it*_ = 2. Thus, we define *Z*_*it*_ = (*X*_*it*_ + *Y*_*it*_)/$\sqrt{2+cor(X_{it},Y_{it})}$ = (*X*_*it*_ + *Y*_*it*_)/1.38. For an uninformative BAF *b*_*it*_ ∉ [0.2,0.8], we define *Z*_*it*_ = *X*_*it*_. Again, when *c*_*it*_ = 2, *Z*_*it*_ ~ *N*(0,1). We then calculate statistic *z*^*i*^_*ab*_ in (1), *U*_*i*+_ in (5) and *p*_*i*+_ in (7) based on the newly defined *Z*_*it*_.

### Implementation and genome-wide scan

The algorithm has been implemented using C++. VTET first normalizes the genome-wide LRRs for each sample to have a zero median and unit variance. VTET tests the CNV associations in a given short genomic region, typically covering 10~100 probes, depending on the probe density and the length of the target genomic region. There are multiple ways to apply VTES to GWAS. For example, we can partition the genome into segments of *M* probes and apply VTET to each of the segments. We can also apply VTET to each gene to perform a gene-based test.

### Simulation studies

To evaluate the statistical performance of VTET, we performed extensive simulations using autosomal SNPs that were present on both the Illumina HumanHap550 SNP array and the Hapmap II SNP list. Our simulations for case-control studies involved two steps: simulating CNV events in subjects and simulating LRRs and BAFs conditioning on the simulated CNV events. Each simulation was based on a given interval with *T* probes. To eliminate the potential impact of minor allele frequencies (MAF) of SNPs in the interval, simulations results were averaged across randomly chosen intervals with *T* probes.

We first describe the procedure to simulate CNV events. Let *f* denote frequency of the risk CNV events in the target genomic interval with *T* probes. Here, the CNV events could be either deletions, or duplications or both. CNVs cover at least *L* (≥3) probes. Let OR be the odds ratio. Then, the frequency of risk CNV events in the case group is given by *f*_+_ = *OR* · *f*/(*OR* · *f* + 1 − *f*). Thus, CNV events were simulated using the Bernoulli distribution with rate *f* for controls and *f*_+_ for cases.

The detailed procedure for simulating LRRs and BAFs for a subject with given copy number status in an interval was described previously (Shi and Li, 2012). Briefly, we randomly drew two haplotypes for the interval from the Hapmap II haplotype pool of European ancestry and specified the copy number status of each SNP in two haplotypes. Then, we simulated LRRs and BAFs for each SNP probe according to the distributions specified in Table 2, which were estimated based on the data produced from Illumina HumanHap550 SNP arrays (Shi and Li, 2012). In summary, our simulations were based on real haplotypes and realistic parameters for LRRs and BAFs. Thus, the results are valuable for the purpose of comparing performance and evaluating the potential for future studies.

**Table 2:** **Parameters characterizing the distribution of LRRs and BAFs**.

**Mean of LRRs**	***SD* of LRRs**	***SD* of BAFs**
(μ _0_,μ _1_,μ _2_,μ _3_,μ _4_)	(σ _0_,σ _1_,σ _2_,σ _3_,σ _4_)	(η _1_,η _2_)
_(−3, −0.45, 0, 0.30, 0.50)_	_(1, 0.26, 0.16, 0.19, 0.22)_	_(0.02, 0.05)_

Given the copy number *c*_*it*_ = k for probe t and subject i, the LRR *X*_*it*_ ~ N(μ_*k*_,σ^2^_*k*_). See Table 1 for the definition of (η_1_,η_2_). The parameters were estimated from the long, experimentally validated CNVs in Illumina 550K arrays.

We compared the statistical power of detecting CNV associations between VTET and the standard two-stage methods. To estimate the power of two-step methods, we first performed simulations to estimate the sensitivity of detecting CNVs for two widely used algorithms, PennCNV (Wang et al., 2007) and CBS (Olshen et al., 2004), using genome-wide intensity data based on Illumina HumanHap550 SNP arrays. We then estimated the power of detecting CNV associations based on the estimated sensitivity for CNV detection. The power was simulated for CNVs covering 3~10 probes.

We also compared the performance of VTET with that of CNVtools (Barnes et al., 2008), an algorithm for testing CNV associations in a given genomic region known with CNV. CNVtools is one of the most efficient algorithms for detecting the association of overlapping or recurrent CNVs. CNVtools first performs principal component analysis (PCA) on the LRRs of all probes in the interval across all subjects and then performs a likelihood ratio test based on the Gaussian mixture model using the first PCA scores. CNVtools requires that the first PCA scores show obvious clustering pattern for different copy number status and will fail without convergence otherwise. When CNVs are recurrent or largely overlapping, CNVtools can succeed in the majority of simulations for deletions and long duplications but not for short duplications. We found that, when CNVs are randomly distributed in the interval, CNVtools fails in almost all simulations.

Thus, we only compared the performance of VTET and CNVtools for recurrent CNVs with identical boundaries. We also compared the power of VTET with the “ideal” power estimated assuming known CNV status. Of note, the power of CNVtools was estimated based on the successful simulations. For example, out of 1000 simulations, CNVtools converges for 800 simulations and detects associations for 500 simulations. The power was estimated as 500/800 = 62.5% instead of 500/1000 = 50%. Thus, the power of CNVTools is overestimated, particularly for short CN3 duplications with non-ignorable failure rates.

### Application to a GWAS of lung cancer

We applied VTET to a GWAS of lung cancer based on the Environment And Genetics in Lung cancer Etiology (EAGLE) study (Landi et al., 2009). Samples were genotyped using the Illumina HumanHap550 SNP arrays. We analyzed ever smokers including 1955 controls and 2374 lung cancer cases. The cases included 587 squamous cell carcinoma (SQ) patients and 920 adenocarcinoma (AD) patients. We partitioned autosomal chromosomes into segments covering 50 SNP probes and tested whether deletions or duplications were overrepresented in cases in each of the segments. We performed an analysis for AD (920 cases and 1955 controls), SQ (587 cases and 1955 controls) and overall lung cancer (2374 cases and 1955 controls) separately. For each test, the *p*-value was accurately estimated by permutations with at least 10 “successful” events.

## Results

### Simulation results for randomly distributed CNVs

CNVs were simulated with the locations randomly distributed in the given interval. Here, simulations were carried out for 2000 subjects (1000 cases and 1000 controls) and 4000 subjects (2000 cases and 2000 controls), intervals with *T* = 20 probes and 40 probes, CNV frequency *f* = 0.01, 0.005, and 0.002, odds ratio *R* = 3, 4, and 5. Power was estimated based on 1000 simulations and different α levels (α = 0.001, 0.01, 0.05). We report the results only for *f* = 0.01 and α = 0.001 because the comparison results for other frequencies and α levels are similar. For each simulation, the *p*-value was calculated based on 10,000 permutations. The ideal power was estimated assuming known CNV status and thus represents the limit of any testing procedure.

**Figure 3:**
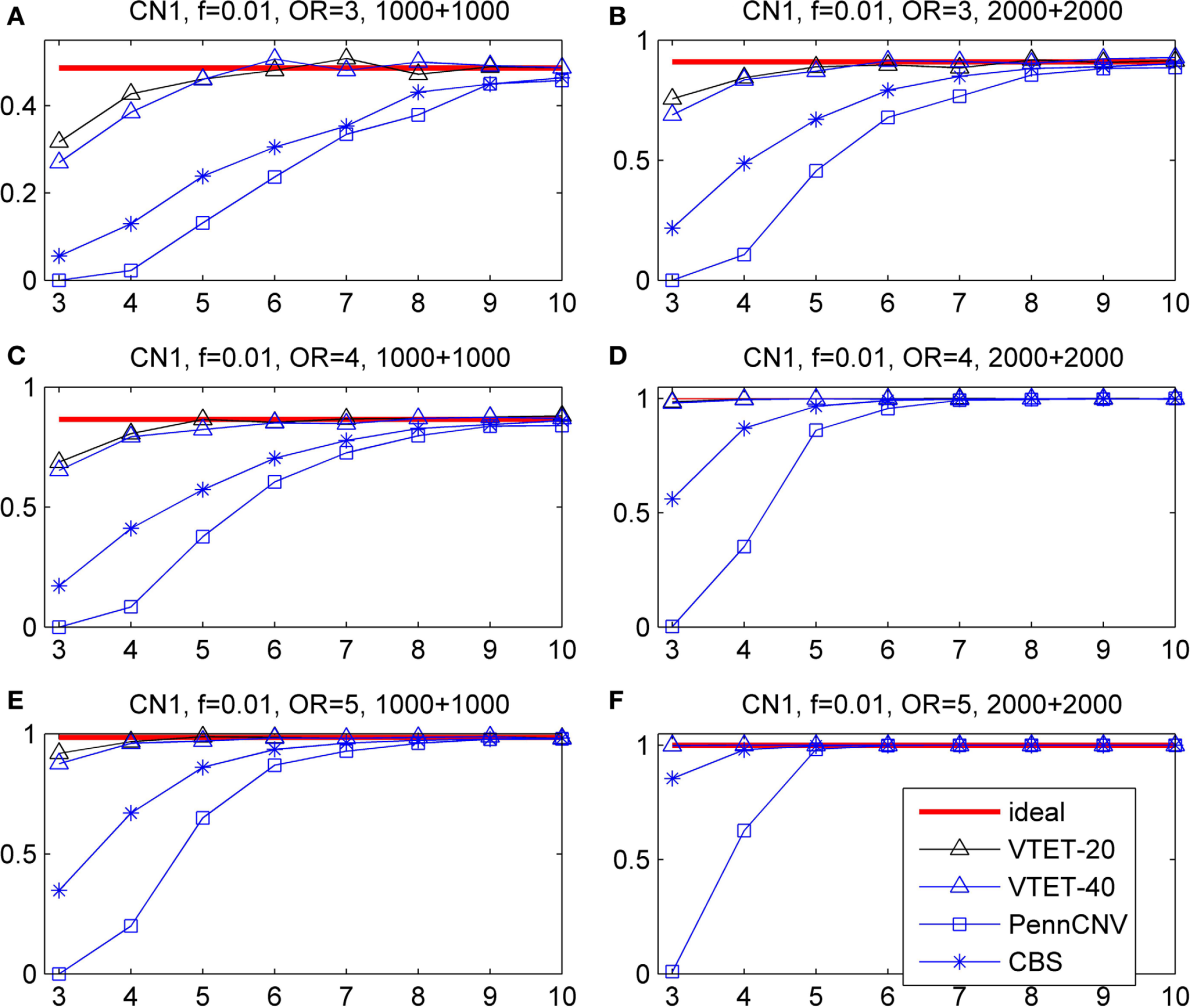
**Statistical power for detecting the association of hemizygous deletions (CN1) with locations randomly distributed in a given region**. *f* is the frequency of CNVs in the general population. OR is odds ratio. The left three panels are based on 1000 cases and 1000 controls. The right three panels are based on 2000 cases and 2000 controls. The x-coordinate is the number of probes covered by the deletions. Powers were calculated based on the significance level α = 0.001. “ideal” denotes the power when CNV status is known and thus represents the limit of the performance of any method. “PennCNV” and “CBS” are the powers from the two-step approach: first calling CNVs using the algorithms and then testing associations based on identified CNVs. The power of PennCNV or CBS does not depend on the length of the interval. “VTET-20 (40)” is the power for detecting CN1 associations in a genomic region with 20 (40) probes using VTET.

**Figure 4:**
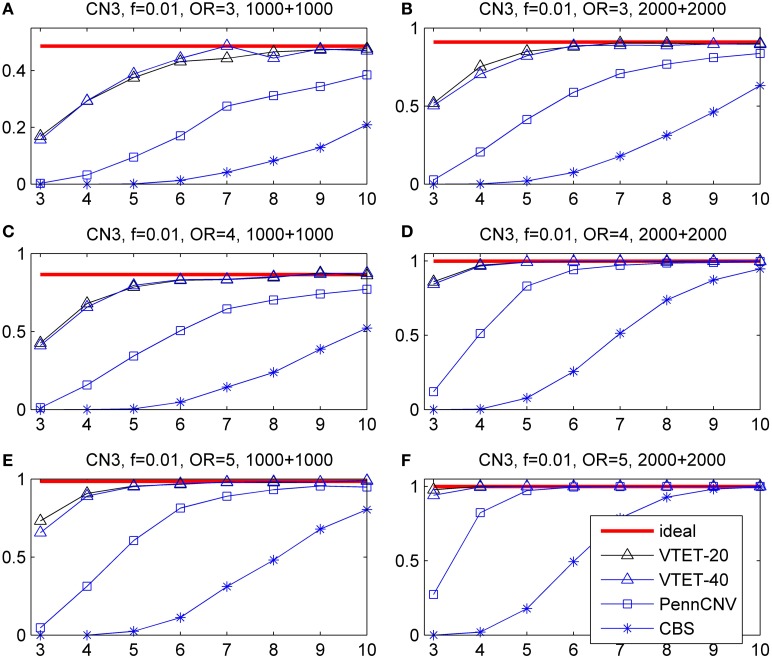
**Statistical power for detecting the association of duplications with three copies (CN3) with locations randomly distributed in a given region**. *f* is the frequency of CNVs in the general population. OR is odds ratio. Powers were calculated based on the significance level α = 0.001. The x-coordinate is the number of probes covered by the duplications. “VTET-20 (40)” is the power for detecting CN3 associations in a genomic region with 20 (40) probes using VTET.

The simulation results are shown in Figure 3 for CN1 hemizygous deletions and Figure 4 for CN3 duplications. We do not report the results for CNVtools because CNVtools failed in almost all simulations (see the explanation in the Materials and Methods section). As expected, power increases with sample size, strength of association measured as OR and the number of probes covered by the CNVs for all testing procedures. In addition, power of VTET depends on the length of the interval. A larger interval implies a larger multiple testing in identifying CNVs and thus typically reduces the power. However, our simulation results suggest that the power of VTET is robust to the length of the tested genomic region.

Compared with the standard two-step testing methods, VTET is more powerful for detecting CNV associations, particularly when CNVs are short. Encouragingly, even for short CNVs, the power of VTET is close to the ideal power estimated assuming known CNV status, suggesting a very high efficiency of VTET.

Note that PennCNV uses both LRRs and BAFs while CBS uses only LRRs. CBS tends to be more sensitive for detecting deletions but less sensitive for detecting duplications from genome-wide intensity data. Thus, as expected, the two-step testing procedure based on PennCNV is more powerful for detecting the association of CN3 duplications but less powerful for CN1 deletions compared to CBS. Of note, the testing procedure based on CBS has no power for detecting the association of short CN3 duplications while the test based on PennCNV has no power for detecting the association of short CN1 deletions.

### Simulation results for recurrent CNVs

While VTET is designed for detecting associations of randomly distributed CNVs, it is important to investigate its performance for recurrent CNVs. Because CNVtools is widely used for detecting associations of recurrent CNVs, we compared VTET with CNVtools for recurrent CNVs. Of note, CNVtools uses only LRRs and cannot use BAFs.

**Figure 5:**
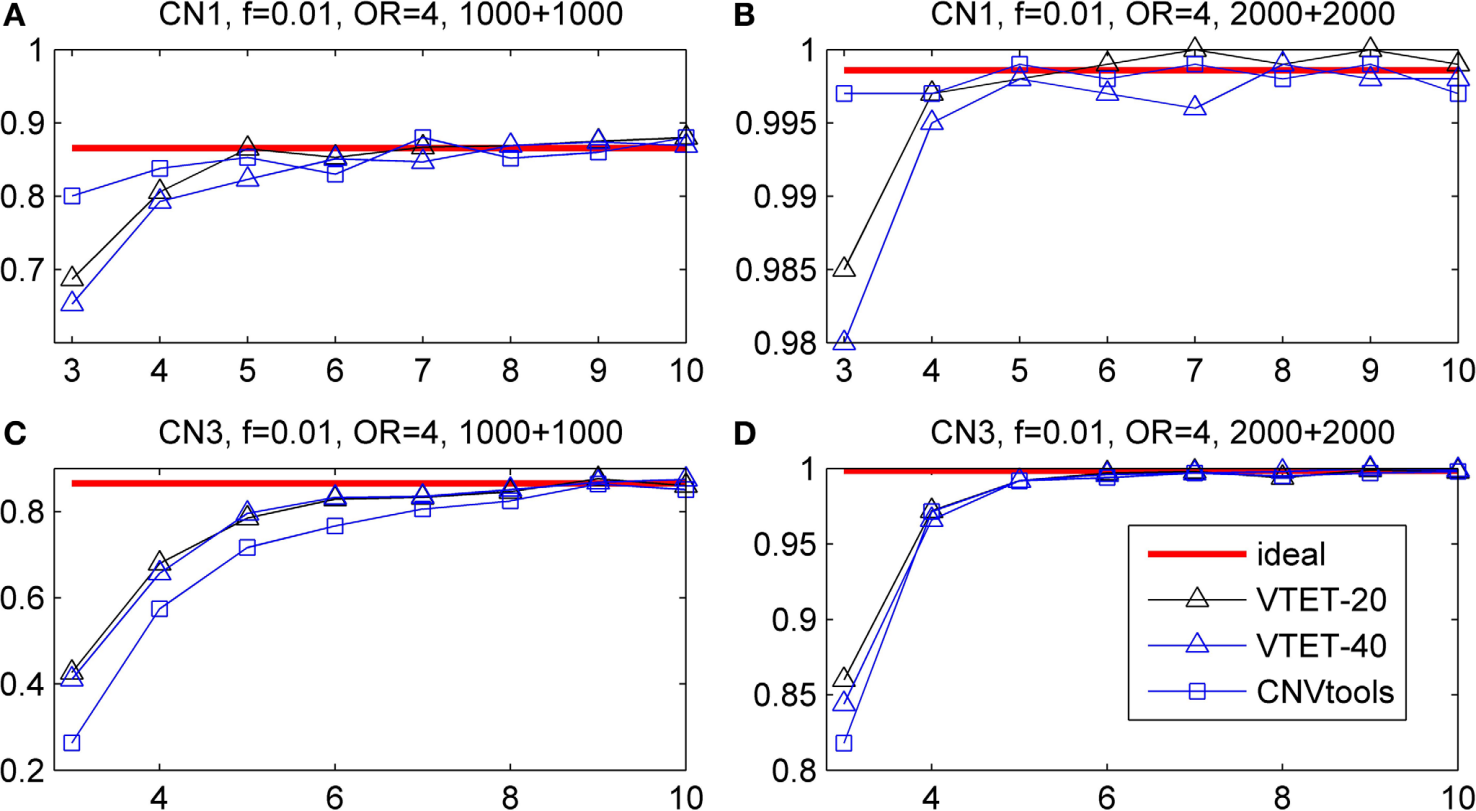
**Statistical power for detecting the association of recurrent CNVs with the same boundary**. *f* is the frequency of CNVs in the general population. OR is odds ratio. Powers were calculated based on the significance level α = 0.001. The x-coordinate is the number of probes covered by the deletions. “ideal” represents the power when CNV status is known. “VTET-20 (40)” is the power for detecting CNV associations in a genomic region with 20 (40) probes using VTET. The power for CNVtools was estimated based on simulations successfully run by CNVtools.

Simulation results are shown in Figure 5. For short CN3 duplications, CNVtools failed in 10% (>6 probes) −30% (3 or 4 probes) of simulations because it could not converge. For short CN1 deletions, CNVtools failed in 1–5% simulations. As expected, failure to explicitly take advantage of the recurrent pattern results in a power loss in VTET, but the power loss is small. CNVtools is slightly more powerful for detecting associations of CN1 deletions. However, VTET is more powerful for detecting associations of CN3 duplications because it uses both LRR and BAF information.

### Results of analyzing eagle lung cancer GWAS

**Figure 6:**
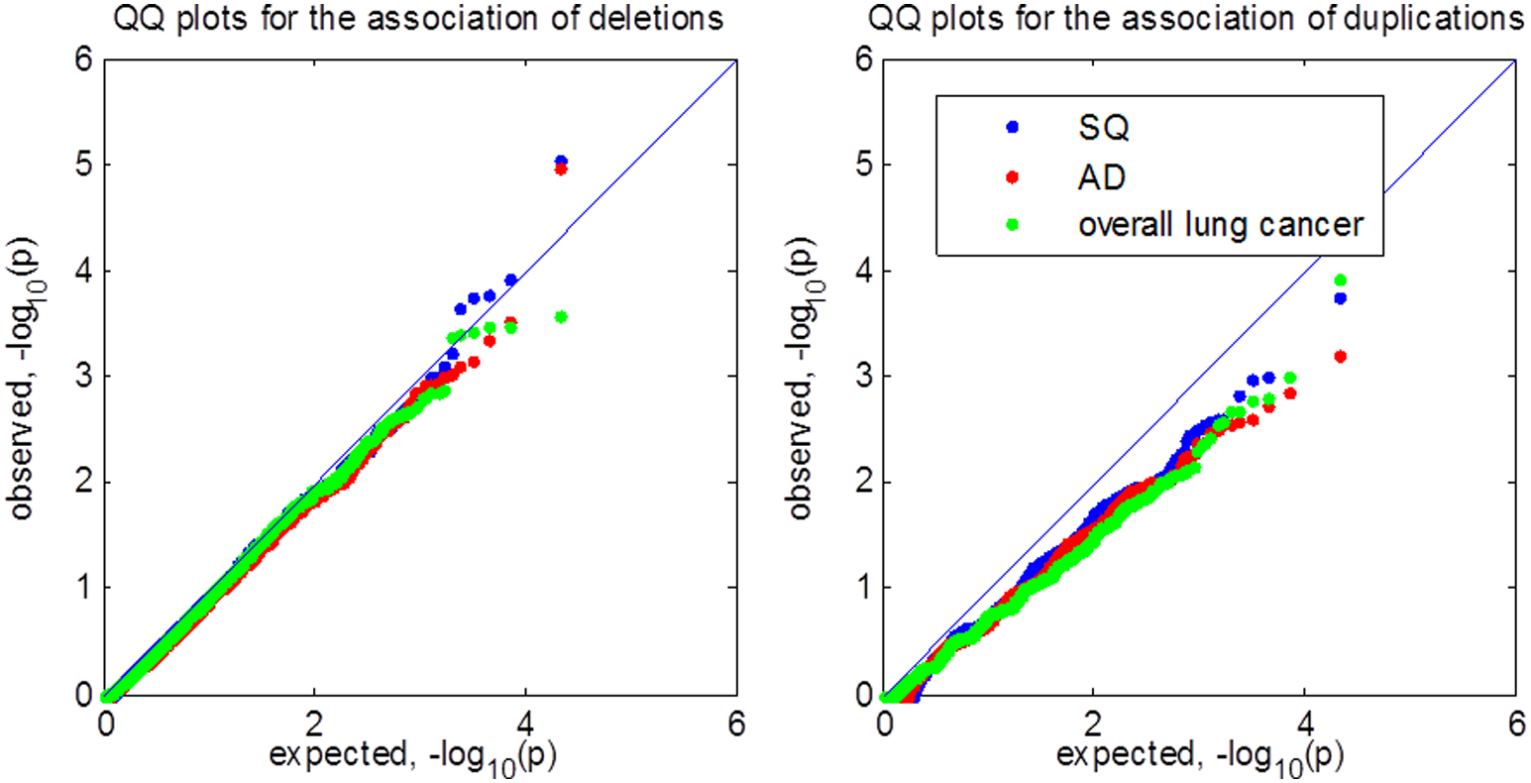
**Quantile-quantile (QQ) plot of *p*-values for testing CNV associations in the EAGLE lung cancer GWAS**. SQ represents lung squamous cell carcinoma. AD represents lung adenocarcinoma. We tested for CNV associations in 10,957 segments covering 50 probes using VTET. All analyses shared the same set of control samples.

We partitioned autosomal chromosomes into 10,957 segments covering 50 probes and applied VTET to each of the segments. The quantile-quantile (QQ) plots for detecting CNV associations in SQ, AD and overall lung cancer are shown in Figure 6. We did not observe a global inflation in any of the analyses, suggesting the validity of VTET. Instead, QQ plots suggest a deflation when analyzing duplications. Further investigation revealed that ~40% of segments had *p*-value = 1 when analyzing duplications while only ~15–20% of segments had *p*-value = 1 when analyzing deletions. This can be explained by the discreteness of the statistics due to the rarity of germline duplications. Typically, deletions are twice more frequent than duplications.

Test statistics are independent across segments. The genome-wide 5% threshold requires *p* = 0.05/10,957 = 4.6 × 10^−6^ based on the Bonferroni correction. No segment reached genome-wide significance under this threshold in any of the analyses. For deletions in SQ, the best *p*-value is 9.0 × 10^−6^ for a segment located at chromosome 18q22.3. Interestingly, in the same segment, the *p*-value for testing the association of duplications is 0.011. When we combined deletions and duplications into one test, the *p*-value for this segment was 4.5 × 10^−6^, reaching genome-wide significance.

## Discussion

Identifying CNVs associated with complex diseases is scientifically important but statistically challenging, particularly for short CNVs because of limited statistical power. Methods have been proposed to directly test associations of recurrent CNVs and have demonstrated superior performance compared to standard two-step testing procedures. In this manuscript, we developed a new method, VTET, for testing associations for CNVs randomly distributed in a short genomic region, a problem that was not addressed by the current methods. We tested this tool in a lung cancer GWAS and have identified a genome-wide significant region on chromosome 18q22.3 for lung squamous cell carcinoma. Lab validation for these tentative CNVs and replication of the association in independent samples are warranted to establish the CNV association with the risk of developing lung squamous cell carcinoma.

VTET utilizes both LRRs and BAFs to maximize the power. We show through simulations that VTET is as powerful as the ideal test for short CNVs covering five or more probes and is only slightly less powerful for shorter CNVs covering three or four probes. In addition, we show that VTET is much more powerful for short CNVs than two-step procedures based on CBS or PennCNV. Recently, methods have been developed for jointly detecting CNVs for multiple samples (Siegmund et al., 2011; Zhang et al., 2012). However, these methods improve the sensitivity only for recurrent CNVs. The two-step testing strategy based on these methods is not expected to improve the power of detecting associations of CNVs randomly distributed in the genomic region. Because VTET does not use spatial information of CNVs, it is not optimal in theory for detecting the association of recurrent CNVs with identical boundaries, under which scenario CNVtools would work the best. However, even under this unfavorable scenario, VTET is only slightly less powerful than CNVtools for short deletions but more powerful for duplications. Thus, VTET can be used for effectively testing the association of both recurrent and non-recurrent CNVs. Finally, we can partition the whole genome into segments flexibly and test for CNV associations using VTET for each segment. We expect that VTET can be used for existing GWAS of complex diseases based on case-control designs.

VTET implicitly assumes that the intensity data, summarized as LRRs and BAFs, have the same distributions in cases and controls. When this assumption is violated, VTET, together with the standard two-step procedures based on CNV calling algorithms, might produce spurious findings, which, in spirit, has been pointed out previously (Barnes et al., 2008). Thus, VTET requires that cases and controls are genotyped using the same genotyping platform and are proportionally balanced in each plate, ideally. VTET is not recommended for studies when cases and controls are genotyped separately, for example, using publically available control data sets. QQ plots are particularly helpful for investigating whether VTET systematically produces spurious findings due to the violation of the assumptions.

Of note, it would useful to extend VTET to next generation sequencing studies, for example whole-exome sequencing studies (WES) and whole-genome sequencing studies (WGS). Again, VTET would implicitly assume that the sequencing depths are similar between cases and controls to avoid spurious findings. It is also useful to extend VTET to meta-analysis of existing GWAS in which the statistical power would be greatly improved. Although meta-analysis for GWAS SNP analysis is straightforward and has been widely investigated, it is more challenging for VTET, both statistically and computationally, particularly when pooling multiple studies with different genotyping platforms. We are currently working on this problem.

In conclusion, VTET can be an important statistical tool to test disease associations of both recurrent and randomly distributed CNVs of various lengths using existing GWAS data.

## Author contrbutions

Peng Li and Jianxin Shi designed the study, implemented the algorithm and performed statistical analyses. Xiaohong R. Yang, Neil E. Caporaso and Maria Teresa Landi participated in the design of the research, interpretation of the results, and contributions to revise the manuscript. Maria Teresa Landi and Neil E. Caporaso are also the PIs of the EAGLE study. Jianxin Shi drafted the manuscript.

### Conflict of interest statement

The authors declare that the research was conducted in the absence of any commercial or financial relationships that could be construed as a potential conflict of interest.
